# Daily changes in fine particulate matter are associated with acute myocardial infarction hospitalizations: an individual-level time-stratified case-crossover study in Zhongshan, China

**DOI:** 10.3389/fpubh.2026.1911027

**Published:** 2026-08-21

**Authors:** Huanling Zeng, Xin Li, Yuhua Zhao, Jiajian Xu, Jinkun Wei

**Affiliations:** 1 Xiaolan Clinical Institute of Shantou University Medical College, Zhongshan, Guangdong, China; 2 Department of Oncology, Xiaolan People’s Hospital of Zhongshan (The Fifth People’s Hospital of Zhongshan), Zhongshan, Guangdong, China; 3 Department of Cardiology, Xiaolan People’s Hospital of Zhongshan (The Fifth People’s Hospital of Zhongshan), Zhongshan, Guangdong, China

**Keywords:** acute myocardial infarction, case-crossover study, daily changes, fine particulate matter, hospitalization

## Abstract

**Background:**

Acute myocardial infarction (AMI) imposes a substantial and growing burden globally. Short-term exposure to ambient fine particulate matter (PM_2.5_) has been linked to AMI mortality, yet most studies have focused on absolute pollutant concentrations. The association between daily changes in PM_2.5_ and AMI hospitalizations remains poorly understood.

**Methods:**

We conducted an individual-level time-stratified case-crossover study in Zhongshan, China, from 2020 to 2024. A total of 1,795 AMI hospitalizations were identified from a tertiary hospital, with 5,877 control days matched by day of week within the same calendar month. Daily PM_2.5_ exposure was assigned based on residential address using the China High Air Pollutants (CHAP) dataset at 1 km resolution. Conditional logistic regression models were fitted to estimate the association between a 10 μg/m^3^ increase in PM_2.5_ (both absolute concentration and day-to-day change) and AMI hospitalization, adjusting for temperature, relative humidity, and public holidays. Subgroup analyses were performed by age, sex, season, AMI subtype, and comorbidities.

**Results:**

A 10 μg/m^3^ increase in PM_2.5_ from the previous day to the admission day (lag0–lag1) was associated with a 7.89% (95% CI: 0.86%–15.40%) higher odds of AMI hospitalization. Associations were found among females (22.58%), adults aged >65 years (22.16%), cold-season admissions (10.85%), and patients with ST-segment elevation myocardial infarction (17.90%), with age identified as a significant effect modifier (*P* for interaction < 0.05). Results remained robust after adjustment for co-pollutants (except for nitrogen dioxide) and alternative degrees of freedom (*df*) for temperature and relative humidity.

**Conclusion:**

Daily increments in ambient PM_2.5_ concentration were independently associated with an increased risk of AMI hospitalization, particularly among females, older adults, and patients with ST-segment elevation myocardial infarction. These findings suggested that day-to-day PM_2.5_ variability may warrant consideration in future cardiovascular prevention frameworks.

## 1 Introduction

Cardiovascular disease (CVD) imposes a substantial global burden of morbidity, mortality, and healthcare utilization. In 2023, CVD accounted for 19.2 million deaths and 437 million disability-adjusted life years (DALYs) worldwide (1). Ischemic heart disease (IHD) constitutes the major component of the CVD burden, and acute myocardial infarction (AMI) represents one of its most severe clinical manifestations (2). In China, the incidence of AMI has risen sharply over recent decades due to population aging, urbanization, and lifestyle transitions, placing increasing strain on healthcare systems (3). Therefore, identifying risk factors of AMI is critically important for developing targeted prevention strategies and reducing the acute burden on medical services.

Among various environmental factors, ambient fine particulate matter (PM_2.5_) has been consistently associated with an increased risk of AMI. In fact, in low- and middle-income countries (LMICs), the mortality burden of cardiovascular diseases attributable to air pollution far exceeds that of smoking and other risk factors (4). However, the vast majority of previous epidemiological studies have focused on AMI mortality (5–7). For instance, a time-stratified case-crossover study of 151,608 a.m. deaths in Hubei, China (2013–2018) found that each 10-μg/m^3^ increment in PM_2.5_ below a breakpoint of 33.3 μg/m^3^ was associated with a 4.14% (95% CI: 1.25–7.12%) increase in AMI mortality, exhibiting a nonlinear exposure–response relationship characterized by a steep slope below the threshold and a plateau at higher concentrations (8). Similarly, another study of 149,632 AMI admissions in Beijing, China (2013–2019) reported that, using a 2-day moving average, each 10-μg/m^3^ increase in PM_2.5_ was associated with a 1.4% (95% CI: 0.9%–1.9%) increase in the risk of in-hospital death (7). While these findings are important, mortality represents only the most severe endpoint and does not capture the full spectrum of AMI burden. Non-fatal outcomes, particularly AMI hospitalizations, are far more common and impose immediate demands on emergency medical systems (9, 10). Despite their direct relevance to healthcare burden and prevention strategies, such hospitalizations have received considerably less attention.

Moreover, existing studies have typically characterized PM_2.5_ exposure using absolute concentration levels, either on the event day alone or as a moving average over several preceding days (11–13). This conventional approach implicitly assumes that the acute cardiovascular effects of PM_2.5_ depend solely on current or recent ambient concentrations, yet it may obscure the health impacts of day-to-day air quality fluctuations. Emerging evidence indicates that exposure variability represents a distinct risk dimension beyond mean concentration. For example, a nationwide study across 251 Chinese cities demonstrated that, at identical mean PM_2.5_ concentrations, mortality risk varied more substantially across different exposure distributions than across differences in mean concentration (14). A Hong Kong study reported that each 10 μg/m^3^ increment in the standard deviation of daily PM_2.5_ was associated with a 1.40% increase in respiratory mortality, independent of the mean concentration (15). Direct evidence also comes from a nationwide case-crossover study of 817,296 patients in China, which introduced air pollution increases between neighboring days (APINs) and found that each interquartile-range increase in PM_2.5_ APINs was associated with a 2.37% (95% CI: 0.88–3.88%) increase in schizophrenia hospitalizations after adjusting for absolute concentrations (16). In fact, a sharp day-to-day increase in PM_2.5_, even within moderate absolute levels, may trigger inflammation, autonomic imbalance, and prothrombotic states, thereby increasing the risk of AMI (17–20). To date, few studies have directly examined whether short-term changes in PM_2.5_ (i.e., the difference between consecutive days) are independently associated with the risk of AMI hospitalization. This knowledge gap impedes the development of refined early warning systems and targeted prevention.

To address these knowledge gaps, we conducted an individual-level time-stratified case-crossover study in Zhongshan, China, to quantify the association between short-term PM_2.5_ exposure and the risk of AMI hospitalization, with a specific focus on the magnitude of daily changes in PM_2.5_ concentrations.

## 2 Methods

### 2.1 Study areas

Zhongshan City, located in the coastal area of Guangdong Province, China, has a land area of approximately 1,781 km^2^ and experiences a humid subtropical monsoon climate with low PM_2.5_ exposure. It is characterized by abundant rainfall, high humidity, and distinct seasonal variations (hot, humid summers and mild, relatively dry winters). The city is also influenced by maritime weather systems, including occasional typhoons during the late summer and early autumn. In our study, electronic medical records were obtained from Xiaolan People’s Hospital, a tertiary-level general hospital in Zhongshan City. Of note, under China’s hospital classification system, tertiary hospitals are designated as the highest tier.

### 2.2 Hospital admissions

Individual-level medical records of AMI inpatients were extracted through a structured electronic medical record system for the period from January 1, 2020, to December 31, 2024. AMI hospitalizations were identified using the International Classification of Diseases, Tenth Revision (ICD-10) code I21 (acute myocardial infarction). The extracted variables included primary diagnosis, date of hospitalization, residential address, sex, age, and comorbidities (hypertension and diabetes). To minimize exposure misclassification and confounding bias due to hospital readmissions, we excluded non-permanent residents of the study city (based on residential address) and all repeat admissions for the same individual. The study protocol was reviewed and approved by the Ethics Committee of Xiaolan People’s Hospital of Zhongshan prior to data extraction (ZSXL-LL2026-044).

### 2.3 Air pollutants and meteorological conditions

Daily ambient concentrations of PM_2.5_, ozone (O_3_), sulfur dioxide (SO_2_), nitrogen dioxide (NO_2_), and carbon monoxide (CO) were obtained from the China High Air Pollutants (CHAP) dataset^1^. This publicly available dataset provides high-resolution, validated estimates of air pollutants at a spatial resolution of 1 km × 1 km. The CHAP data have been extensively validated and widely applied in epidemiological studies across China, supporting their robustness for air pollution exposure assessment (21–23). In our study, individual-level air pollutant exposures were assigned based on participants’ residential addresses. Specifically, each residential address was geocoded to obtain longitude and latitude coordinates, and daily exposure estimates were derived by matching these coordinates to the corresponding raster grid cells for specific dates.

Daily mean temperature and relative humidity data were sourced from the Resource and Environmental Science Data Platform^2^, operated by the Chinese Academy of Sciences’ Institute of Geographic Sciences and Natural Resources Research. Individual-level meteorological exposures were assigned by linking each participant’s residential address to the closest monitoring station.

### 2.4 Statistical analyses

We used a time-stratified case-crossover design and fitted conditional logistic regression models. For each patient with AMI, the case day was defined as the date of hospital admission. For each case, we selected three or four control days, matching on the same day of the week within the same calendar month and year. This approach inherently controls for long-term trends, seasonality, and time-invariant individual-level confounders (e.g., genetic predisposition, chronic comorbidities, and socioeconomic status) (24–26). Two specifications of the exposure were considered: (1) the daily average PM_2.5_ concentration, and (2) the difference in PM_2.5_ concentration between the current day and the preceding day. Models were adjusted for daily mean temperature and relative humidity using natural cubic splines (3 degrees of freedom each) (27, 28), as well as for public holidays. The conditional logistic regression model is specified by:


$$\begin{array}{l} \text{Logit}\;P\;(\text{case}=1\;\text{in stratum}\;i)=\text{αstratum}\;i\\ +\beta \;(\mathrm{PM}2.5\;\text{or daily changes in}\;\mathrm{PM}2.5)\\ +\mathrm{ns}\;(\text{temperature},\mathrm{df}=3)\\ +\mathrm{ns}\;(\text{relative humidity},\mathrm{df}=3)+\text{holiday} \end{array}$$

*P* denotes the conditional probability that a given day within stratum *i* serves as the case day, where each stratum comprises one case day and its corresponding control days matched within the same individual. The parameter *β* represents the main exposure effect, quantifying the change in the odds of AMI hospitalization associated with either a 10 μg/m^3^ increment in the daily average PM_2.5_ concentration or the day-to-day variation in PM_2.5_. Consistent with prior case-crossover studies, smoothing of meteorological confounders was achieved using natural cubic splines, denoted as *ns*(), with 3 degrees of freedom assigned to both daily mean temperature and relative humidity (12, 29). Additionally, a binary covariate, *Holiday*, was included to indicate whether a date falls on a nationally designated public holiday in China. We applied the conditional likelihood ratio test to evaluate the fit of the conditional logistic regression model. To verify whether the constrained model (with day-to-day PM_2.5_ variability) provides a reasonable simplification of the data, AIC (Akaike Information Criterion) and BIC (Bayesian Information Criterion) were used to compare the goodness-of-fit between the constrained model and the unconstrained model (with lag0 and lag1 PM_2.5_ as separate predictors). Percentage changes were calculated as (odds ratio [OR]−1) × 100.

The temporal relationship was explored using two lag structures: (1) single-day lags from lag0 (admission day) to lag6 (the previous 6 days), and (2) moving average exposures over periods of lag0–1 through lag0–6 (30, 31). To evaluate the secondary hypothesis concerning rapid PM_2.5_ fluctuations, we derived day-to-day absolute changes in PM_2.5_ concentration (e.g., the difference between lag1 and lag0, lag2 and lag1, and so forth up to lag6 and lag5) and assessed their associations with AMI hospital admissions.

We examined effect modification by stratifying on age, sex, admission season (warm: April to September; cold: October to March), myocardial infarction subtype (non-ST-segment elevation myocardial infarction, NSTEMI or ST-segment elevation myocardial infarction, STEMI), pre-existing comorbidities (hypertension and diabetes) and COVID-19 pandemic period. To further examine the independence of the day-to-day PM_2.5_ variability effect, we additionally performed subgroup analyses stratified by the median of case-day PM_2.5_ exposure concentrations. Differences in effect estimates between subgroups were assessed using the Z-test, with the test statistic defined as $(E_{1}-E_{2})\pm \sqrt{S{E_{1}}^{2}+S{E_{2}}^{2}}$ , where *E* and *SE* represent the effect estimate and its standard error for each subgroup. To assess the robustness of our primary findings, we conducted sensitivity analyses by adding each co-pollutant (O_3,_ SO_2_, NO_2_, and CO) individually into the models for daily average PM_2.5_ concentration and for daily changes in PM_2.5_. We further tested the sensitivity to degrees of freedom for temperature and relative humidity by varying *df* from 2 to 5. All statistical analyses were conducted using Python (version 3.10) and R (version 4.2.0). Statistical significance was defined as a two-tailed *p* value < 0.05.

## 3 Results

### 3.1 Study population characteristics, PM_2.5_ concentrations, and meteorological conditions

This study included a total of 1,795 AMI case days and 5,877 matched control days from 2020 to 2024 (Supplementary Table S1). Among AMI cases, the majority were male (80.3%) and aged ≤65 years (70.4%). Admissions were evenly distributed between warm and cold seasons, and NSTEMI accounted for 74.5% of cases. The prevalence of hypertension and diabetes was 41.6% and 66.3%, respectively. Distributions of air pollutant concentrations and meteorological variables were comparable between case and control days (Supplementary Table S2). The median PM_2.5_ level was 19.5 μg/m^3^ (IQR: 11.9–29.7) on case days versus 19.0 μg/m^3^ (IQR: 11.6–28.7) on control days. Temperature and relative humidity did not differ substantially between the two groups. The Spearman correlation matrix among air pollutants and meteorological variables on case days was presented in Supplementary Table S3.

### 3.2 Association of PM_2.5_ with AMI hospitalizations

**Figure 1:**
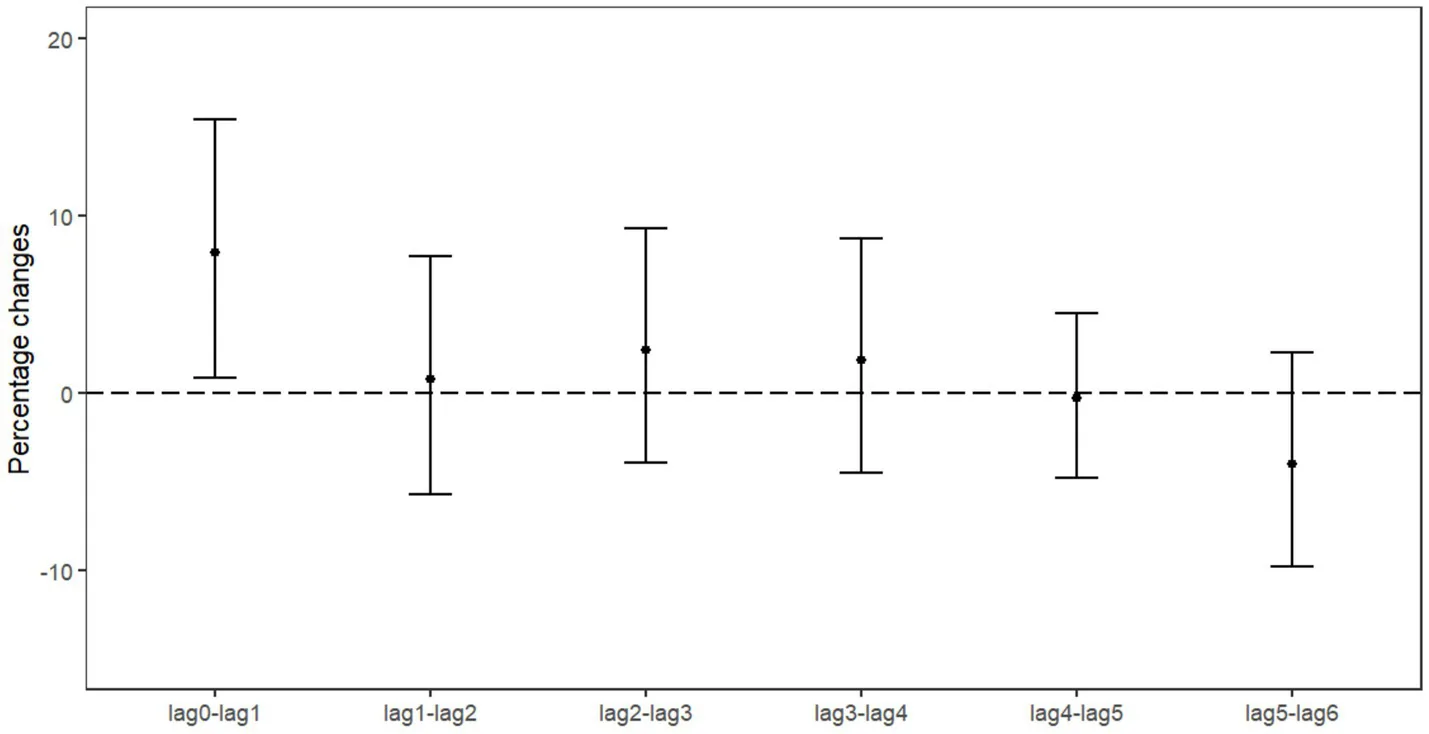
Percentage changes in the risk of AMI admissions associated with each 10 μg/m^3^ daily change in PM_2.5_ concentration. AMI, acute myocardial infarction; CI, confidence interval; lag0, the day of AMI admission; lag1, the previous day; lag2, the previous 2 days; lag3, the previous 3 days; lag4, the previous 4 days; lag5, the previous 5 days; lag6, the previous 6 days.

**Figure 2:**
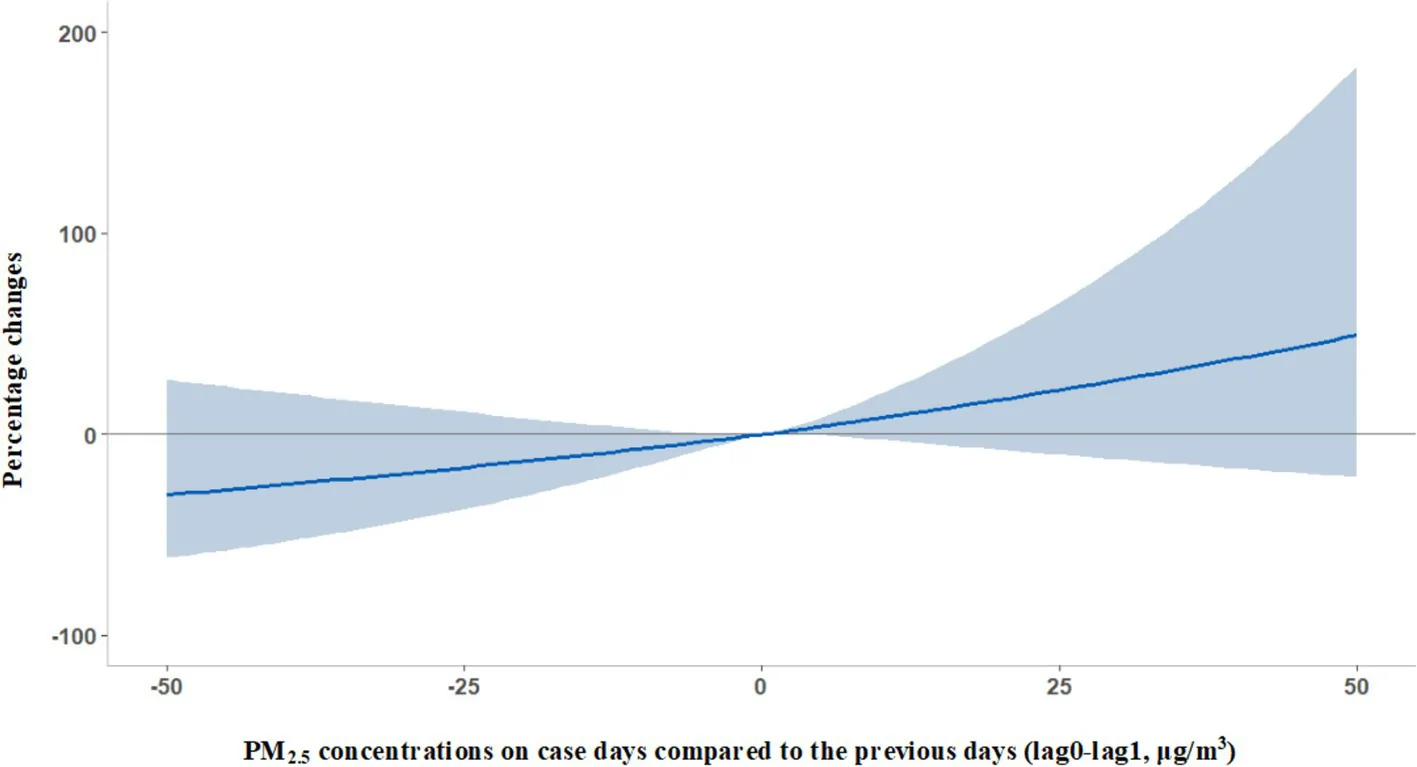
Exposure–response curves for the association between daily changes in PM_2.5_ concentrations on case days compared to the previous days (lag0-lag1) and percentage changes in the risk of AMI admissions. AMI, acute myocardial infarction; lag0, the day of AMI admission; lag1, the previous day.

In single-day lag models, a 10 μg/m^3^ increase in PM_2.5_ concentration on the day of admission (lag0) was associated with a 5.86% higher risk of AMI hospitalization (95% CI: 0.00–12.10; *p* < 0.05) (Supplementary Table S4, Figure S1). No statistically significant associations were observed for other single-day lags (lag1 through lag6) or for moving-average exposure windows (lag0-1 through lag0-6). Regarding the change in PM_2.5_ from the previous day, a 10 μg/m^3^ increase was associated with a 7.89% elevation in AMI risk (95% CI: 0.86–15.40; *p* < 0.05) (Supplementary Table S5, Figure 1). The conditional likelihood ratio test yielded a statistically significant result (*χ*^2^ = 19.4, *p* < 0.05), indicating that the fitted model provided a satisfactory explanation of the data. The constrained model (with day-to-day PM_2.5_ variability) yielded an AIC of 5192.97 and a BIC of 5236.92, while the unconstrained model (with lag0 and lag1 PM_2.5_ as separate predictors) yielded an AIC of 5193.84 and a BIC of 5243.27, indicating that the constrained model achieved a marginally better fit. Other between-day comparisons did not reach statistical significance. Exposure-response curves demonstrated a generally monotonic increase in AMI risk with rising PM_2.5_ concentrations at lag0 and with acute changes in PM_2.5_ exposure (lag0–lag1) (Figure 2, Supplementary Figure S2).

### 3.3 Stratified analyses

Subgroup analyses revealed differential susceptibility across populations (Table 1, Supplementary Table S6). For acute PM_2.5_ increase (lag0-lag1), significantly elevated risks were observed among females (22.58%; 95% CI: 4.87–43.30; *p* < 0.05), adults aged >65 years (22.16%; 95% CI: 7.77–38.50; *p* < 0.05), cold-season admissions (10.85%; 95% CI: 2.58–19.80; *p* < 0.05), and patients with STEMI (17.90%; 95% CI: 2.68–35.40; *p* < 0.05). A significant interaction was identified for age group in the lag0-lag1 model (*p* < 0.05). Meanwhile, for same-day exposure (lag0), a significant association was observed only during the cold season (8.99%; 95% CI: 2.14–16.30; *p* < 0.05). When stratified by case-day PM₂.₅ exposure concentration, the effect was significant in the group with concentrations above the median level of 19.5 μg/m^3^ (11.34, 95% CI: 2.40–21.07, *p* < 0.05).

**Table 1:** Percentage changes in the risk of AMI admissions associated with each 10 μg/m^3^ daily change in PM_2.5_ exposure on case days compared to the previous days (lag0-lag1).

Subgroup	Percentage change	95% CI	*p*-value	*P* for interaction
Sex				0.08
Male	4.65	(−2.91, 12.80)	0.235	
Female	22.58	(4.87, 43.30)	**<0.05**	
Age group				**<0.05**
≤65y	2.60	(−5.33, 11.20)	0.531	
>65y	22.16	(7.77, 38.50)	**<0.05**	
Admission season				0.197
Warm	0.50	(−12.92, 15.90)	0.950	
Cold	10.85	(2.58, 19.80)	**<0.05**	
Type of myocardial infarction				0.138
NSTEMI	4.79	(−3.02, 13.20)	0.236	
STEMI	17.90	(2.68, 35.40)	**<0.05**	
Hypertension				0.394
Yes	8.47	(−1.19, 19.10)	0.087	
No	7.34	(−2.65, 18.30)	0.155	
Diabetes				0.369
Yes	10.05	(−3.36, 25.30)	0.149	
No	6.70	(−1.40, 15.50)	0.108	
Case-day PM_2.5_ exposure concentration				0.199
<Median level (19.5 μg/cm^3^)	1.91	(−9.69, 15.00)	0.759	
≥Median level (19.5 μg/cm^3^)	11.34	(2.40, 21.07)	<0.05	
COVID-19 pandemic period				0.360
During (2020–2022)	6.55	(−2.00, 15.90)	0.137	
After (2023–2024)	10.08	(−1.73, 23.30)	0.097	

AMI, acute myocardial infarction; CI, confidence interval; lag0, the day of AMI admission; lag0, the day of AMI admission; lag1, the previous day; warm season, April to September; cold season, October to March. Bold values indicate statistically significant results (*p* < 0.05).

### 3.4 Sensitivity analyses

After adjusting for gaseous co-pollutants, the associations between PM_2.5_ and AMI risk remained generally robust (Table 2, Supplementary Table S7). For lag0–lag1, significant associations persisted after adjusting for O_3_, SO_2_, and CO (estimates: 8.92%–9.04%; all *p* < 0.05), but were attenuated to non-significance following NO_2_ adjustment (5.96%; *p* = 0.120). No significant interactions with co-pollutants were detected. Sensitivity analyses with different *df* settings for temperature and humidity produced PM_2.5_ estimates consistent with the main model (Table 3).

**Table 2:** Percentage changes in the risk of AMI admissions associated with each 10 μg/m^3^ daily change in PM_2.5_ exposure on case days compared to the previous days (lag0-lag1) with additional adjustment of SO_2_, NO_2_ and CO.

Model	Percentage change	95% CI	*p*-value	*P* for interaction
Main model	7.89	(0.86, 15.40)	**<0.05**	Reference
+O_3_	8.92	(1.61, 16.80)	**<0.05**	0.392
+SO_2_	8.25	(0.80, 16.20)	**<0.05**	0.398
+NO_2_	5.96	(−1.50, 14.00)	0.120	0.374
+CO	9.04	(1.63, 17.00)	**<0.05**	0.390

AMI, acute myocardial infarction; CI, confidence interval; lag0, the day of AMI admission; lag1, the previous day. Bold values indicate statistically significant results (*p* < 0.05).

**Table 3:** Percentage changes in the risk of AMI admissions associated with each 10 μg/m^3^ daily change in PM_2.5_ exposure on case days compared to the previous days (lag0-lag1) under alternative alternative degrees of freedom (*df*) for temperature and relative humidity.

Model	Percentage change	95% CI	*p*-value	*P* for interaction
Main model (*df* = 3)	7.89	(0.86, 15.40)	**<0.05**	Reference
*df* = 2	7.81	(0.81, 15.30)	**<0.05**	0.399
*df* = 4	7.62	(0.60, 15.10)	**<0.05**	0.398
*df* = 5	7.64	(0.60, 15.20)	**<0.05**	0.398

AMI, acute myocardial infarction; CI, confidence interval; lag0, the day of AMI admission; lag1, the previous day. Bold values indicate statistically significant results (*p* < 0.05).

## 4 Discussion

In this individual-level time-stratified case-crossover study conducted in Zhongshan, China, we found that short-term daily changes in ambient PM_2.5_ were significantly and positively associated with AMI hospitalizations. A 10 μg/m^3^ elevation in PM_2.5_ from the pre-admission day to the admission day (lag0-lag1) was associated with a 7.89% (95% CI: 0.86–15.40) increase in the odds of AMI hospitalization. Stratified analyses revealed stronger associations among females, older adults (>65 years), cold-season admissions, and STEMI patients, with age group identified as a significant effect modifier (*P* for interaction < 0.05). To the best of our knowledge, this is the first individual-level case-crossover study in China to specifically quantify the association between day-to-day changes in PM_2.5_ and AMI hospitalizations, providing novel evidence that acute fluctuations in PM_2.5_ may serve as an independent risk factor of AMI events.

Most prior epidemiological studies on air pollution and AMI have focused on absolute PM_2.5_ concentrations (32–34). For instance, an updated umbrella review of systematic reviews and meta-analyses revealed that cumulative evidence demonstrated a robust association between short-term PM_2.5_ exposure and increased risk of myocardial infarction (RR: 1.02, 95% CI: 1.01–1.03) (11). Another systematic review and meta-analysis, which pooled 123 effect estimates from 100 epidemiological studies, found that short-term PM_2.5_ exposure was associated with a pooled risk ratio of 1.0090 (95% CI: 1.0074–1.0106) for cardiovascular mortality per 10 μg/m^3^ increment (6). While these studies consistently reported positive associations with AMI mortality or hospitalization, they largely overlooked the potential impact of rapid day-to-day changes in air quality. Recent evidence, however, has begun to challenge the adequacy of relying solely on mean concentrations as exposure metrics. A nationwide study across 251 Chinese cities demonstrated that, at identical mean PM_2.5_ concentrations, mortality risk exhibited greater variation across different exposure distributions than across differences in mean concentration, suggesting that the temporal pattern of exposure, rather than its average level alone, may critically influence health outcomes (14). This epidemiological evidence is further complemented by two additional studies (15, 16). Nevertheless, no prior study has directly examined whether such day-to-day increases in PM_2.5_ are independently associated with the risk of AMI. Our study extends this literature by demonstrating that except for absolute PM_2.5_ levels, a sharp increase in PM_2.5_ from the previous day confers an additional, independent risk of AMI hospitalization, suggesting that dynamic changes in environmental exposures may trigger acute cardiovascular events through mechanisms distinct from those of sustained exposure. Furthermore, compared with studies examining AMI mortality, our focus on hospitalizations captures a more common and immediately resource-intensive endpoint. In China, AMI incidence has risen sharply over recent decades due to population aging and lifestyle transitions (35, 36), placing increasing strain on emergency medical services. Thus, identifying triggers of non-fatal AMI events is of direct public health and health system relevance. Notably, our study was conducted in a low-PM_2.5_ setting (median: 19.5 μg/m^3^). In fact, 93.6% of the PM_2.5_ concentrations on the case day (lag0) and the preceding day (lag1) were below 50 μg/m^3^, the daily average limit for Grade II areas under the latest China Ambient Air Quality Standard (GB 3095–2026), reinforcing the conclusion that no safe threshold for PM_2.5_ exists and the day-to-day variability effect is possibly an independent risk indicator beyond high absolute exposure levels.

Furthermore, it should be noted that the association between day-to-day PM_2.5_ variability and AMI hospitalization is likely to be asymmetric, meaning that the effect magnitude may differ between concentration increases and decreases. This is consistent with the nonlinear exposure-response relationship between PM_2.5_ and cardiovascular disease documented in previous studies (6, 37). Rather than dichotomizing the data into concentration increase and decrease groups, which may introduce methodological bias because days with concentration decreases tend to correspond to higher baseline absolute concentrations while days with increases often arise from lower baseline levels, thereby confounding the effect of direction with that of absolute concentration, this study provides an overall estimate of the association between day-to-day PM_2.5_ variability and AMI hospitalization risk. Future studies with larger sample sizes and more refined time-series data are warranted to further examine and quantify the differential health effects of this asymmetry.

The biological plausibility of the observed association between rapid increases in PM_2.5_ and AMI hospitalization can be explained by three interconnected pathways. First, even at relatively low concentrations, PM_2.5_ exposure can rapidly reduce heart rate variability, suggesting that autonomic nervous system imbalance represents a highly sensitive early-response pathway (38, 39). Second, PM_2.5_ exposure induces oxidative stress and systemic inflammation, promoting vascular endothelial cell apoptosis and plaque instability (40). Third, PM_2.5_ constituents, particularly organic carbon and black carbon, have been shown to mediate blood pressure elevation and prothrombotic effects through the arachidonic acid metabolic pathway, with exposure-response curves exhibiting a linear, threshold-free pattern (41). Taken together, these mechanisms suggest that a sharp increase in PM_2.5_ concentration may itself constitute an acute physiological stress that surpasses the compensatory capacity of susceptible individuals, thereby triggering acute coronary events independently of absolute concentration levels.

The observed association between acute PM_2.5_ increases and AMI hospitalizations is biologically plausible. Rapid rises in PM_2.5_ concentration have been shown to induce systemic inflammatory responses, characterized by elevated circulating levels of interleukins (e.g., IL-6) and C-reactive protein (42, 43). These inflammatory mediators can destabilize coronary atherosclerotic plaques, rendering them prone to rupture (44). In addition, short-term PM_2.5_ exposure is known to impair cardiac autonomic function, as reflected by reduced heart rate variability, which may predispose to arrhythmias and ischemic events (45). Prothrombotic effects, including increased platelet activation and fibrinogen levels, further contribute to acute coronary thrombosis (46, 47). While few studies have directly investigated the association between rapid PM_2.5_ elevations and AMI, available evidence nevertheless lends biological plausibility to this relationship.

Our stratified analyses revealed differential susceptibility across populations, providing insights into potential mechanisms and high-risk groups. Older adults (>65 years) exhibited significantly stronger associations for lag0–lag1 PM_2.5_ changes compared with younger individuals, and age was identified as a significant effect modifier (*P* for interaction < 0.05). This may be explained by age-related declines in physiological reserve, higher baseline inflammation, increased prevalence of comorbidities, and reduced adaptive capacity to environmental stressors (48, 49). Females also showed a pronounced risk elevation (22.58%; 95% CI: 4.87–43.30), which is consistent with some previous studies (50, 51). Potential explanations include smaller coronary artery diameter, greater autonomic reactivity, or hormonal interactions with inflammatory pathways (52), though further research is needed. The stronger association observed during the cold season (10.85%; 95% CI: 2.58–19.80) is consistent with known synergies between low temperature and PM_2.5_ exposure. Cold exposure can induce vasoconstriction, increase blood pressure, and enhance the pro-inflammatory effects of particulate matter, thereby amplifying cardiovascular risk (53, 54). The differential effect by AMI subtype—STEMI patients showing a larger risk estimate (17.90, 95% CI: 2.68–35.40) than NSTEMI patients—suggests that acute PM_2.5_ increases may preferentially trigger complete coronary occlusion, possibly through more pronounced prothrombotic or vasospastic mechanisms (55). Finally, in stratified analyses comparing the COVID-19 pandemic (2020–2022) and post-pandemic (2023–2024) periods, effect estimates were directionally consistent with the primary model but did not reach statistical significance, possibly owing to limited sample size in the post-pandemic period and healthcare system disruptions during the pandemic. Of note, all subgroup analyses were exploratory in nature and were not pre-specified prior to the initiation of this study. Given that multiple comparisons may inflate the Type I error rate, statistically significant findings from the subgroup analyses should be interpreted as hypothesis-generating rather than hypothesis-confirming. Additionally, in stratified analyses the *p* values for interaction did not reach statistical significance for most subgroups (with the exception of age group) and the limited sample size in some subgroups may also have compromised statistical power. Therefore, these subgroup differences should be interpreted with caution, and the findings should be viewed as exploratory rather than definitive and their clinical and public health implications warrant further validation in future hypothesis-driven studies.

Notably, in sensitivity analyses with additional adjustment for NO_2_, the effect estimate for the association between day-to-day PM_2.5_ variability and AMI admission risk decreased from 7.89% to 5.96% and lost statistical significance (*p* = 0.120). However, this attenuation does not negate the public health relevance of day-to-day PM_2.5_ variability as an independent risk indicator. Several potential explanations may account for this observation. First, NO₂ is a major marker of traffic-related emissions and may exhibit substantial correlation with PM_2.5_ (Spearman’s *ρ* = 0.79 in the present study). As both pollutants share similar emission sources (particularly motor vehicle exhaust) (56), adjusting for NO_2_ may statistically absorb the portion of the PM_2.5_ effect attributable to the mixed traffic-related exposure, thereby attenuating the estimate. Second, NO₂ itself may exert independent cardiovascular toxicity (57), and its collinearity with PM_2.5_ makes it methodologically challenging to fully disentangle their independent contributions. Third, the possibility that NO_2_ acts as a confounder cannot be entirely excluded, as it may affect cardiovascular health through other pathways, such as inducing oxidative stress and systemic inflammation, rather than serving merely as a marker of co-pollutant exposure. These findings suggest that the effect of day-to-day PM_2.5_ variability may be partially attributable to mixed traffic-related exposure. Future studies are warranted to investigate the differential contributions of PM_2.5_ components to AMI risk, with the aim of more precisely identifying the key constituents of traffic emissions that are truly cardiotoxic.

Although the case-crossover design has advantages in controlling for individual-level confounding, given the observational design of this study, establishing causality requires further prospective studies and mechanistic investigations. With this caveat in mind, our findings offer the following exploratory implications for public health practice. First, most of current air quality early warning systems rely predominantly on absolute PM_2.5_ concentration thresholds. Our findings suggest that day-to-day PM_2.5_ variability may warrant consideration. Second, the subgroups identified as potentially more susceptible in our exploratory analyses might benefit from precautionary advice, such as limiting outdoor exposure and using personal protective measures on days with pronounced PM_2.5_ increases, even when absolute levels are not exceptionally high. Third, it is conceivable that forecasting these fluctuations could offer useful lead information for emergency services and hospitals to better prepare for possible short-term increases in AMI admissions, though this would need to be evaluated in future operational studies.

This study has several strengths. First, using residential geocoding and high-resolution CHAP data for individual-level exposure assessment reduced exposure misclassification relative to area-level methods. Second, the time-stratified case-crossover design inherently accounted for time-invariant individual confounders (e.g., genetics, chronic conditions, and socioeconomic status), as well as for long-term trends and seasonality. Finally, and importantly, our focus on day-to-day PM_2.5_ fluctuations represents a novel exposure metric that has seldom been examined in association with AMI hospitalizations.

Nevertheless, several limitations should be acknowledged. First, this single-center study was conducted in Zhongshan, a coastal city in Guangdong Province in China with low PM_2.5_ levels and a subtropical monsoon climate. Its population and healthcare settings may differ from inland or northern regions. Thus, generalization of our findings to areas with distinct PM₂.₅ profiles, climates, or healthcare systems requires caution. Multi-center studies are needed to confirm external validity. Second, PM_2.5_ exposure was assigned based on residential addresses without accounting for individual activity patterns (indoor/outdoor time, commuting, workplace exposure), which may have introduced misclassification and conservative estimates. However, the self-matching design of case-crossover studies minimizes short-term changes in activity patterns, thereby partially mitigating this bias. Future research integrating personal mobility data and indoor/outdoor monitoring would enhance exposure accuracy. Third, we did not explore potential spatial heterogeneity in the PM_2.5_ effect across different regions or by proximity to major roads, which may also have introduced non-differential misclassification and biased the estimates toward the null (58, 59). Future studies could incorporate land-use regression (LUR) models or traffic volume data to further investigate the modifying effects of spatial variations in air pollution distribution. Fourth, meteorological exposures were assigned via proximity to the nearest monitoring station, rather than using spatial interpolation methods. Despite Zhongshan’s small area (1,781 km^2^) and flat terrain, which limit spatial temperature variability, this approach may still introduce measurement error in confounders, potentially attenuating PM_2.5_ effect estimates and yielding conservative results. Fifth, we adopted single-day lag and pairwise change analyses rather than Distributed Lag Model (DLM) or Distributed Lag Nonlinear Model (DLNM). While day-to-day variability poses challenges for DLM/DLNM, future studies may adopt advanced approaches to better capture the cumulative and delayed effects of PM_2.5_ changes. Finally, although the case-crossover design controls for time-invariant confounders (e.g., genetics, comorbidities, smoking, and socioeconomic status), several time-varying factors, such as influenza outbreaks, seasonal medication adherence, and occupational exposure were not accounted for and may have introduced residual confounding.

## 5 Conclusion

This individual-level case-crossover study in Zhongshan, China, demonstrates that day-to-day increases in PM_2.5_ concentration are independently associated with a higher risk of AMI hospitalization, even in a low-pollution setting. The association was stronger among older adults, females, cold-season admissions, and STEMI patients. These findings extend the traditional focus on absolute pollutant levels and suggest that acute fluctuations in PM_2.5_ should be incorporated into air quality health warning systems and clinical prevention strategies. Future multi-center studies with personal exposure monitoring and detailed time-activity data are warranted to validate and extend our findings.

## Data Availability

The raw data supporting the conclusions of this article will be made available by the authors, without undue reservation.

## Footnotes

1
https://weijing-rs.github.io/product.html

2
https://www.resdc.cn
