# Supplementary material for: Daily changes in fine particulate matter are associated with acute myocardial infarction hospitalizations: an individual-level time-stratified case-crossover study in Zhongshan, China
Source: Front Public Health. 2026 Aug 21;14:1911027. doi: 10.3389/fpubh.2026.1911027 (PMC13541734; doi:10.3389/fpubh.2026.1911027)
Supplement: Supplementary file 1 [file Data_Sheet_1.docx]

**Supplementary materials**

**Supplementary Table S1.** Summary statistics and characteristics of AMI cases

**Supplementary Table S2.** Summary distribution of air pollutant concentrations and meteorological conditions on case days and control days

**Supplementary Table S3.** Spearman correlation matrix among air pollutants and meteorological variables

**Supplementary Table S4.** Percentage changes in the risk of AMI admissions associated with each 10 μg/m^3^ increase in PM_2.5_ exposure

**Supplementary Table S5.** Percentage changes in the risk of AMI admissions associated with each 10 μg/m^3^ daily change in PM_2.5_ concentration

**Supplementary Table S6.** Percentage changes in the risk of AMI admissions associated with each 10 μg/m^3^ increase in PM_2.5_ exposure on case days (lag0)

**Supplementary Table S7.** Percentage changes in the risk of AMI admissions associated with each 10 μg/m^3^ increase in PM_2.5_ exposure on case days (lag0) with additional adjustment of SO_2_, NO_2_ and CO

**Supplementary Figure S1.** Percentage changes in the risk of AMI admissions associated with each 10 μg/m^3^ increase in PM_2.5_ exposure

**Supplementary Figure S2.** Exposure-response curves for the association between PM_2.5_ concentrations at lag0 and percentage changes in the risk of AMI admissions

**Supplementary Table S1.** Summary statistics and characteristics of AMI cases

|  | Number | Percentage (%) |
| --- | --- | --- |
| **Total** | 1795 | 100 |
| **Sex** |  |  |
| Male | 1441 | 19.72 |
| Female | 354 | 80.28 |
| **Age group** |  |  |
| ≤ 65y | 1264 | 70.42 |
| > 65y | 531 | 29.58 |
| **Admission season** |  |  |
| Warm | 896 | 49.92 |
| Cold | 899 | 50.08 |
| **Type of myocardial infarction** |  |  |
| NSTEMI | 1337 | 74.48 |
| STEMI | 458 | 25.52 |
| **Hypertension** |  |  |
| Yes | 973 | 41.62 |
| No | 822 | 49.27 |
| **Diabetes** |  |  |
| Yes | 1310 | 66.33 |
| No | 485 | 24.56 |

Abbreviations: AMI, acute myocardial infarction.

Warm season: from April to September; Cold season: October to March

**Supplementary Table S2.** Summary distribution of air pollutant concentrations and meteorological conditions on case days and control days

|  | **Median** | **（25th, 75th）** |
| --- | --- | --- |
| **On case days (n=1795)** |  |  |
| PM_2.5_ (μg/m^3^) | 19.5 | (11.9, 29.7) |
| PM_2.5_ (lag0-lag1) (μg/m^3^) | 0.2 | (-2.9, 3.9) |
| Temperature (℃) | 24.9 | (19.8, 28.4) |
| Relative humidity (%) | 78 | (71, 85) |
| O_3_ (μg/m^3^) | 86.7 | (62.1, 126.6) |
| SO_2_ (μg/m^3^) | 6.7 | (5.8, 7.9) |
| NO_2_ (μg/m^3^) | 25.8 | (17.6, 36.9) |
| CO (mg/m^3^) | 0.7 | (0.6, 0.8) |
| **On control days (n=5877)** |  |  |
| PM_2.5_ (μg/m^3^) | 19.0 | (11.6, 28.7) |
| PM_2.5_ (lag0-lag1) (μg/m^3^) | 0.2 | (-3.3, 3.5) |
| Temperature (℃) | 24.8 | (19.7, 28.4) |
| Relative humidity (%) | 78 | (71, 85) |
| O_3_ (μg/m^3^) | 85.9 | (62.0, 128.1) |
| SO_2_ (μg/m^3^) | 6.6 | (5.8, 7.9) |
| NO_2_ (μg/m^3^) | 25.2 | (18.0, 35.7) |
| CO (mg/m^3^) | 0.7 | (0.6, 0.8) |

Abbreviations: lag0, the day of acute myocardial infarction admission; lag1, the previous day.

**Supplementary Table S3.** Spearman correlation matrix among air pollutants and meteorological variables on case days and PM_2.5_ exposure on case days compared to the previous days (lag0-lag1)

|  | Tem | RH | PM_2.5_ | O_3_ | NO_2_ | SO_2_ | CO | PM_2.5_ (lag0-lag1) |
| --- | --- | --- | --- | --- | --- | --- | --- | --- |
| Tem | 1.00 | 0.28^*^ | -0.46^*^ | 0.22^*^ | -0.56^*^ | -0.34^*^ | -0.52^*^ | -0.08^*^ |
| RH | 0.28^*^ | 1.00 | -0.37^*^ | -0.42^*^ | -0.27^*^ | -0.61^*^ | -0.04^*^ | -0.14^*^ |
| PM_2.5_ | -0.46^*^ | -0.37^*^ | 1.00 | 0.43^*^ | 0.79^*^ | 0.71^*^ | 0.71^*^ | 0.33^*^ |
| O_3_ | 0.22^*^ | -0.42^*^ | 0.43^*^ | 1.00 | 0.16^*^ | 0.46^*^ | 0.14^*^ | 0.19^*^ |
| NO_2_ | -0.56^*^ | -0.27^*^ | 0.79^*^ | 0.16^*^ | 1.00 | 0.69^*^ | 0.74^*^ | 0.26^*^ |
| SO_2_ | -0.34^*^ | -0.61^*^ | 0.71^*^ | 0.46^*^ | 0.69^*^ | 1.00 | 0.48^*^ | 0.25^*^ |
| CO | -0.52^*^ | -0.04^*^ | 0.71^*^ | 0.14^*^ | 0.74^*^ | 0.48^*^ | 1.00 | 0.18^*^ |
| PM_2.5_  (lag0-lag1) | -0.08^*^ | -0.14^*^ | 0.33^*^ | 0.19^*^ | 0.26^*^ | 0.25^*^ | 0.18^*^ | 1.00 |

Abbreviations: Tem, daily mean temperature; RH, relative humidity; lag0, the day of acute myocardial infarction admission; lag1, the previous day.

^*^ *P* <0.05

**Supplementary Table S4.** Percentage changes in the risk of AMI admissions associated with each 10 μg/m^3^ increase in PM_2.5_ exposure

| Lag | Percentage Change | 95% CI | *P* value |
| --- | --- | --- | --- |
| lag0 | 5.86 | (0.00, 12.10) | **<0.05** |
| lag1 | 0.19 | (-5.48, 6.20) | 0.949 |
| lag2 | -0.35 | (-5.81, 5.40) | 0.902 |
| lag3 | -2.12 | (-7.42, 3.50) | 0.451 |
| lag4 | -3.24 | (-8.33, 2.10) | 0.231 |
| lag5 | -2.81 | (-7.84, 2.50) | 0.293 |
| lag6 | 0.01 | (-5.10, 5.40) | 0.996 |
| lag0–1 | 3.76 | (-2.67, 10.60) | 0.258 |
| lag0–2 | 2.74 | (-4.12, 10.10) | 0.443 |
| lag0–3 | 1.34 | (-5.87, 9.10) | 0.723 |
| lag0–4 | -0.18 | (-7.63, 7.90) | 0.964 |
| lag0–5 | -1.38 | (-9.44, 7.40) | 0.750 |
| lag0–6 | -1.38 | (-9.97, 8.00) | 0.766 |

Abbreviations: AMI, acute myocardial infarction; CI, confidence interval; lag0, the day of AMI admission; lag1, the previous day; lag2, the previous 2 days; lag3, the previous 3 days; lag4, the previous 4 days; lag5, the previous 5 days; lag6, the previous 6 days; lag0–1, moving average of the case day and the previous day; lag0–2, moving average of the case day and the previous 2 days; lag0–3, moving average of the case day and the previous 3 days; lag0–4, moving average of the case day and the previous 4 days; lag0–5, moving average of the case day and the previous 5 days; lag0–6, moving averages of the case day and 1 to 6 preceding days.

**Supplementary Table S5.** Percentage changes in the risk of AMI admissions associated with each 10 μg/m^3^ daily change in PM_2.5_ concentration

| Lag | Percentage Change | 95% CI | *P* value |
| --- | --- | --- | --- |
| lag0-lag1 | 7.89 | (0.86, 15.40) | **<0.05** |
| lag1-lag2 | 0.74 | (-5.74, 7.70) | 0.827 |
| lag2-lag3 | 2.45 | (-3.95, 9.30) | 0.462 |
| lag3-lag4 | 1.88 | (-4.51, 8.70) | 0.573 |
| lag4-lag5 | -0.27 | (-4.79, 4.50) | 0.910 |
| lag5-lag6 | -3.98 | (-9.83, 2.30) | 0.206 |

Abbreviations: AMI, acute myocardial infarction; CI, confidence interval; lag0, the day of AMI admission; lag1, the previous day; lag2, the previous 2 days; lag3, the previous 3 days; lag4, the previous 4 days; lag5, the previous 5 days; lag6, the previous 6 days.

**Supplementary Table S6.** Percentage changes in the risk of AMI admissions associated with each 10 μg/m^3^ increase in PM_2.5_ exposure on case days (lag0)

| Subgroup | Percentage Change | 95% CI | *P* value | *P* for interaction |
| --- | --- | --- | --- | --- |
| **Sex** |  |  |  | 0.283 |
| Male | 4.52 | (-1.93, 11.40) | 0.173 |  |
| Female | 11.00 | (-2.22, 26.00) | 0.107 |  |
| **Age group** |  |  |  | 0.339 |
| ≤ 65y | 4.64 | (-2.33, 12.10) | 0.197 |  |
| > 65y | 8.44 | (-2.06, 20.10) | 0.119 |  |
| **Admission season** |  |  |  | 0.121 |
| Warm | -2.55 | (-14.11, 10.60) | 0.688 |  |
| Cold | 8.99 | (2.14, 16.30) | **<0.05** |  |
| **Type of myocardial infarction** |  |  |  | 0.372 |
| NSTEMI | 5.16 | (-1.57, 12.40) | 0.136 |  |
| STEMI | 7.81 | (-3.63, 20.60) | 0.189 |  |
| **Hypertension** |  |  |  | 0.393 |
| Yes | 6.48 | (-1.47, 15.10) | 0.113 |  |
| No | 5.42 | (-3.07, 14.60) | 0.218 |  |
| **Diabetes** |  |  |  | 0.323 |
| Yes | 9.34 | (-2.35, 22.40) | 0.122 |  |
| No | 4.70 | (-1.98, 11.80) | 0.172 |  |

Abbreviations: AMI, acute myocardial infarction; CI, confidence interval; lag0, the day of AMI admission; warm season, April to September; cold season, October to March.

**Supplementary Table S7.** Percentage changes in the risk of AMI admissions associated with each 10 μg/m^3^ increase in PM_2.5_ exposure on case days (lag0) with additional adjustment of SO_2_, NO_2_ and CO

| **Model** | **Percentage Change** | **95% CI** | ***P* value** | ***P* for interaction** |
| --- | --- | --- | --- | --- |
| **Main model** | 5.86 | (0.00, 12.10) | **<0.05** | reference |
| **+O_3_** | 10.31 | (2.96, 18.18) | **<0.05** | 0.266 |
| **+SO_2_** | 8.81 | (0.95, 17.30) | **<0.05** | 0.385 |
| **+NO_2_** | 3.25 | (-4.49, 11.60) | 0.421 | 0.184 |
| **+CO** | 10.31 | (2.75, 18.40) | **<0.05** | 0.399 |

Abbreviations: AMI, acute myocardial infarction; CI, confidence interval; lag0, the day of AMI admission.

**Supplementary Figure S1.** Percentage changes in the risk of AMI admissions associated with each 10 μg/m^3^ increase in PM_2.5_ exposure

Abbreviations: AMI, acute myocardial infarction; lag0, the day of AMI admission; lag1, the previous day; lag2, the previous 2 days; lag3, the previous 3 days; lag4, the previous 4 days; lag5, the previous 5 days; lag6, the previous 6 days; lag0–1, moving average of the case day and the previous day; lag0–2, moving average of the case day and the previous 2 days; lag0–3, moving average of the case day and the previous 3 days; lag0–4, moving average of the case day and the previous 4 days; lag0–5, moving average of the case day and the previous 5 days; lag0–6, moving averages of the case day and 1 to 6 preceding days.

**Supplementary Figure S2.** Exposure-response curves for the association between PM_2.5_ concentrations at lag0 and percentage changes in the risk of AMI admissions

Abbreviations: AMI, acute myocardial infarction; lag0, the day of AMI admission.
